# Supplementary material for: Splice-Junction-Based Mapping of Alternative Isoforms in the Human Proteome
Source: Cell Rep. Author manuscript; Available in PMC 2020 Jan 15. (PMC6961840; doi:10.1016/j.celrep.2019.11.026)
Supplement: 2 [file NIHMS1546469-supplement-2.zip › DF1/PXD009737/TestisLysC/TestisLysC_17_GIGYF1_LMPLGPSCLSRYLPEWLRALSGGGSVASPPPSPAMPK.pdf]

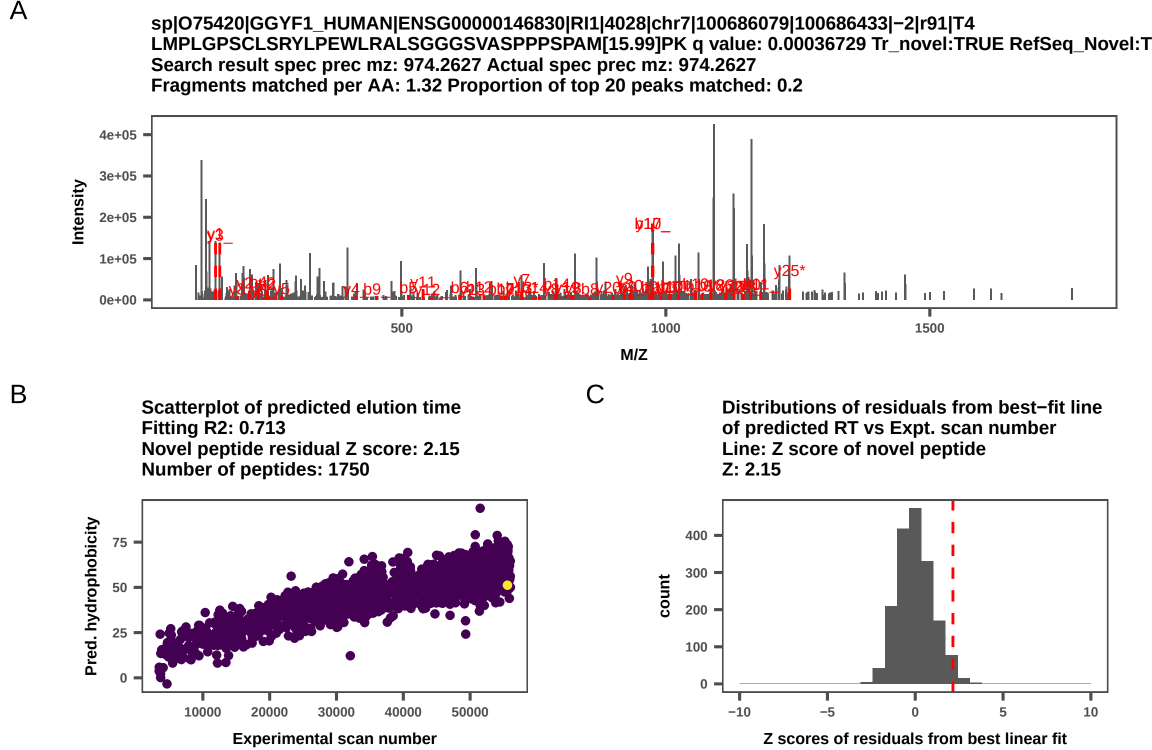
A
sp|O75420|GGYF1_HUMAN|ENSG00000146830|RI1|4028|chr7|100686079|100686433|−2|r91|T4
LMPLGPSCLSRYLPEWLRALSGGGSVASPPPSPAM[15.99]PK q value: 0.00036729 Tr_novel:TRUE RefSeq_Novel:T
Search result spec prec mz: 974.2627 Actual spec prec mz: 974.2627
Fragments matched per AA: 1.32 Proportion of top 20 peaks matched: 0.2
Intensity
4e+05
3e+05
2e+05
1e+05
0e+00
y25*
500
1000
1500
M/Z
B
Scatterplot of predicted elution time
Fitting R2: 0.713
Novel peptide residual Z score: 2.15
Number of peptides: 1750
Pred. hydrophobicity
75
50
25
0
10000
20000
30000
40000
50000
Experimental scan number
C
Distributions of residuals from best−fit line
of predicted RT vs Expt. scan number
Line: Z score of novel peptide
Z: 2.15
count
400
300
200
100
0
−10
−5
0
5
10
Z scores of residuals from best linear fit
